# Induction of Low Temperature Tolerance in Wheat by Pre-Soaking and Parental Treatment with Melatonin

**DOI:** 10.3390/molecules26041192

**Published:** 2021-02-23

**Authors:** Hua Zhang, Lei Liu, Zongshuai Wang, Guozhong Feng, Qiang Gao, Xiangnan Li

**Affiliations:** 1 College of Resources and Environment, Jilin Agricultural University, Changchun 130102, China; 15764379737@163.com (H.Z.); gyt199962@163.com (Q.G.); 2 Key Laboratory of Mollisols Agroecology, Northeast Institute of Geography and Agroecology, Chinese Academy of Science, Changchun 130102, China; liulei@iga.ac.cn; 3 Crop Research Institute, Shandong Academy of Agricultural Sciences, Jinan 250100, China; wzshuai0109@163.com

**Keywords:** melatonin, *Triticum aestivum*, low temperature, germination, carbohydrate metabolism

## Abstract

Low temperatures seriously depress germination and seedling establishment in wheat and it is of great significance to explore approaches to improve wheat tolerance to low temperatures. In this study, the effects of seed pre-soaking and parental treatment with melatonin on seed germination and low temperature tolerance during the early growing stage in wheat were studied. The results showed that pre-soaking with melatonin increased the germination rate, improved antioxidant capacity and accelerated starch degradation under low temperature, which alleviated low temperature-induced damage to the chloroplasts in coleoptiles of wheat seedlings. Parental melatonin treatment during grain filling stage significantly decreased the grain weight. Seeds from parental melatonin-treated plants showed higher germination rates and higher antioxidant enzyme activity than the control seeds under low temperature. In addition, parental treatment with melatonin modulated the activities of carbohydrate metabolism enzymes, which contributes to enhanced low temperature tolerance in wheat offspring. It was suggested that both seed pre-soaking and parental treatment with melatonin could be the effective approaches for low temperature tolerance induction in wheat.

## 1. Introduction

Low temperatures are one of the most prominent environmental stresses which greatly limits crop plant development and grain yield [1]. Growth of winter wheat (*Triticum aestivum* L.) is extremely vulnerable to low temperatures [2]. Wheat germination is easily limited by low temperatures due to depressed metabolism activity [3]. In germinating seeds, the reduction in amylase activity induced by low temperatures results in lower degradation of seed storage reserves, such as starch, hence limiting the energy supply available for seed germination [4]. Meanwhile, low temperatures disturb the antioxidant systems [3], leading to a obvious reduction in the reactive oxygen species (ROS) scavenging capacity [5]. Low temperatures increase the membrane viscosity of the photosynthetic apparatus and thylakoid electron transport, leading to a lower photosynthetic carbon assimilation efficiency [6]. The enzymes involved in carbohydrate metabolism are also very sensitive to temperature changes, which is directly related to the plant growth [7].

As an important signal molecule, melatonin (*N*-acetyl-5-methoxytryptamine) is involved in almost all processes in plants [8], such as seed germination [9], plant growth [10] and grain filling [11]. A large body of evidence has shown that melatonin plays an important role in the adaptation of plants to abiotic stress, including drought [12], waterlogging [13], heat [14], salt stress [15] and low temperature exposure [16]. Melatonin-induced stress tolerance is associated with the activation of antioxidant defense systems [17]. For instance, exogenously applied melatonin enhances the antioxidant capacity in both chloroplasts and mitochondria, which sustains the photosynthetic electron transport in the photosynthetic apparatus in barley under low temperature conditions [18]. Foliar melatonin application during recovery promotes the cold priming-induced tolerance to subsequent low temperatures in wheat [19]. In addition, the ameliorative effects of melatonin on drought stress in grape cuttings has been illustrated [20]. Under cold stress, melatonin treatment increases the concentration of proline, which helps to protect wheat seedlings from cold stress [21].

Numerous studies have indicated that the changes induced by former stress events or exogenous agents could be inherited by offspring plants [22,23]. Parental ABA treatment during the grain filling stage increases the activities of antioxidant enzymes in germinating seeds and leaves, hence favoring establishment of offspring seedlings in wheat under low temperature stress [24]. Parental jasmonic acid treatment induced heritable modifications of DNA methylation which results in changes in the offspring transcriptome and metabolome in dandelion [25]. In addition, our previous study documented that spraying with melatonin promotes the seed germination of offspring wheat through enhancing amylase activity and shortening germination time [26]. However, the mechanisms of induction of low temperature tolerance by presoaking and parental treatment with melatonin are still barely known.

Thus, these two melatonin treatments were applied to the germinating seeds and the growing seeds in the parental plants during the grain filling phase. The seed germination and seedling establishment under low temperature conditions were tested. It was hypothesized that: (i) presoaking seeds with melatonin improves the seed germination under low temperatures; (ii) the parental melatonin treatment during grain filling would enhance the cold tolerance in offspring wheat.

## 2. Results

### 2.1. Effects of Seed Pre-Soaking with Melatonin on Seed Germination under Low Temperature

**Figure 1:**
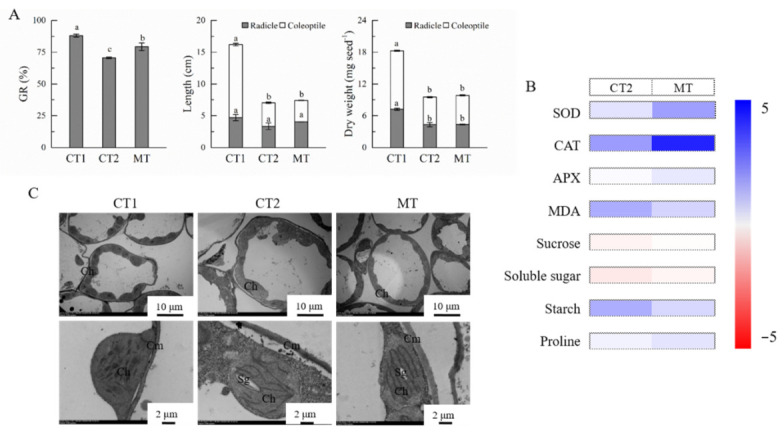
Effects of pre-soaked with exogenous melatonin on wheat germination under low temperature. (**A**), effects of pre-soaked with exogenous melatonin on seed germination rate (GR), length of radicle and coleoptile, and dry weight of radicle and coleoptile after 7 days′ germination under low temperature in wheat. Different small letters (a, b, and c) mean significant difference between treatments at *p* < 0.05 level. (**B**), heatmap of antioxidant enzyme activity and metabolite concentration in germinating seeds as affected by exogenous melatonin under low temperature. The difference of antioxidant enzyme activity and metabolite concentration among these treatments is deviation standardization and converted to a color scale. Increase and decrease of antioxidant enzyme activity and metabolite concentration are indicated in the colorbar. SOD, superoxide dismutase; CAT, catalase; APX, ascorbate peroxidase; MDA, malondialdehyde. (**C**), TEM images of chloroplasts in the coleoptile of germinating seeds pre-soaked with exogenous melatonin. Top row images: bar = 10 μm; bottom row images: bar = 2 μm. Ch, chloroplast; Cm, cell membrane; Sg, starch grain. CT1, normal temperature control; CT2, low temperature control; MT, melatonin treatment under low temperature. Data are expressed as mean ± SE (*n* = 3).

The germination rate, the length and dry weight of radicle and coleoptile were all significantly reduced by low temperatures compared with those under normal temperature conditions (CT1, Figure 1A). The germination rate in the melatonin treatment (MT) group was significantly higher than that in the low temperature control (CT2). No significant difference was found in either length or dry weight of radicle and coleoptiles between MT and CT2.

The activities of antioxidant enzymes and concentration of malondialdehyde (MDA) were higher under low temperature conditions than those under normal temperature (Figure 1B, Figure S1). Seed pre-soaking with melatonin significantly improved the activity of superoxide dismutase (SOD) under low temperature compared with CT2. A similar trend was found in the activities of catalase (CAT) and ascorbate peroxidase (APX). In addition, the MDA concentration in MT was significantly lower than that in CT2.

The concentrations of sucrose and soluble sugar were the highest while starch concentration was the lowest in CT1 among all treatments (Figure 1B, Figure S1). Under low temperature conditions, melatonin pre-soaking significantly reduced the starch concentration in germinating seeds, while it increased the concentrations of sucrose and soluble sugar, in relation to CT2. In addition, melatonin pre-soaking enhanced the proline concentration under low temperature (Figure 1B, Figure S1).

After 7 days′ germination, fusiform chloroplasts were observed in the coleoptile close to the cell wall with a clear and complete outer membrane under normal temperature conditions (Figure 1C). The basal grains and stroma lamella were arranged in parallel along the long axis of the chloroplasts. However, the development of chloroplasts was significantly affected by low temperature exposure. The shape of swelling chloroplasts was changed from fusiform to a round shape under low temperature. The outer membrane of chloroplasts was separated from the cell wall, while the basal grains and stroma lamella were not completely formed. Under low temperature conditions, the outer membrane of chloroplasts in MT was located much closer to the cell wall than those in CT2.

### 2.2. Effects of Parental Melatonin Treatment on Germination of Offspring under Low Temperature.

**Figure 2:**
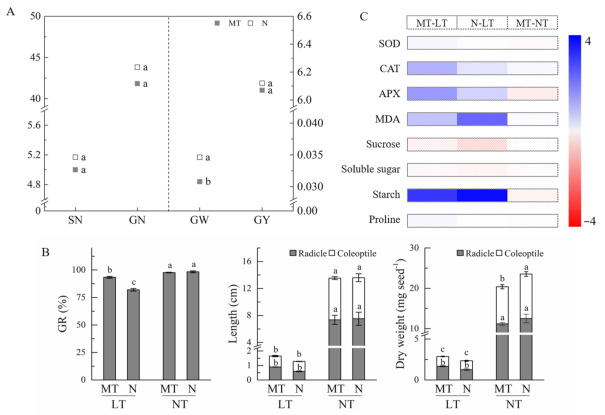
Effects of parental melatonin treatment during grain filling on offspring wheat germination under low temperature. (**A**), effects of spraying with melatonin during grain filling stage on grain yield of wheat at maturity. SN, spike number per plant; GN, grain number per spike; GW, grain weight; GY, grain yield per plant. Different small letters (a, b, and c) mean significant difference between treatments at *p* < 0.05 level. (**B**), effects of parental melatonin treatment during grain filling on seed germination rate (GR), length of radicle and coleoptile, and dry weight of radicle and coleoptile after 7 days′ germination of offspring wheat under low temperature. Different small letters mean significant difference between treatments at *p* < 0.05 level. (**C**), heatmap of antioxidant enzyme activity and metabolite concentration in offspring wheat germinating seeds as affected by parental melatonin treatment and low temperature. The difference of antioxidant enzyme activity and metabolite concentration among these treatments is deviation standardization and converted to a color scale. Increase and decrease of antioxidant enzyme activity and metabolite concentration is indicated in the colorbar. SOD, superoxide dismutase; CAT, catalase; APX, ascorbate peroxidase; MDA, malondialdehyde. MT, melatonin treatment; N, the control; LT, low temperature; NT, normal temperature. Data are expressed as mean ± SE (*n* = 3).

At maturity, grain weight was significantly reduced by melatonin treatment compared with the control (Figure 2A). Spike number per plant, grain number per spike and grain yield per plant were not significantly affected by melatonin treatment.

The seed germination rate was significantly depressed by low temperature (Figure 2B, Table S1). However, parental melatonin treatment significantly increased the germination rate under low temperatures in relation to the control. In addition, the parental melatonin treatment had no significant effect on either length or dry weight of radicle and coleoptile under low temperature conditions.

Activities of antioxidant enzymes under low temperature were significantly higher than those under NT (Figure 2C, Table S1, Figure S2). However, no significant difference in SOD activity in germinating seeds was found between MT and N under low temperature. Activities of CAT and APX were remarkably enhanced by MT treatment compared with the control under low temperature. In addition, the increase in MDA concentration induced by low temperature was lower in MT seeds than that in N seeds.

Low temperature lowered the starch degradation and increased the concentrations of sucrose and soluble sugar in seeds compared with normal temperature (Figure 2C, Table S1, Figure S2). Under low temperature conditions, parental melatonin treatment significantly increased sucrose concentration while it decreased starch concentration, in relation to the control. However, it had no significant effect on the concentration of soluble sugar under low temperature. In addition, the proline concentration was not affected by either MT or low temperature (Figure 2C, Table S1, Figure S2).

### 2.3. Effects of Parental Melatonin Treatment during Grain Filling on Offspring Wheat Seedling under Low Temperature.

**Figure 3:**
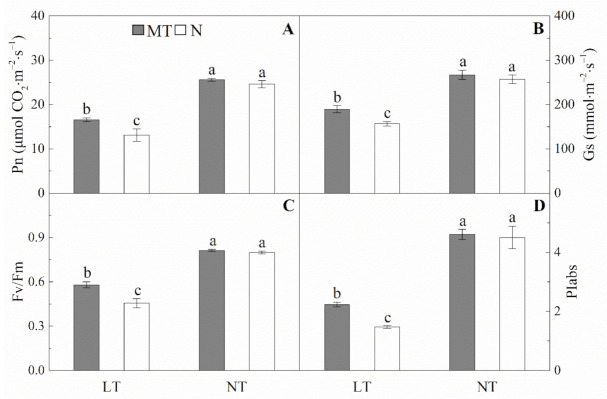
Effects of parental melatonin treatment during grain filling on Pn (**A**), Gs (**B**), Fv/Fm (**C**) and PIabs (**D**) in the last fully expanded leaf of offspring wheat seedlings under low temperature. Different small letters (a, b, and c) mean significant difference between treatments at *p* < 0.05 level. MT, melatonin treatment; N, the control; LT, low temperature; NT, normal temperature. Pn, net photosynthetic rate; Gs, stomatal conductance; Fv/Fm, maximum quantum efficiency of photosystem Ⅱ; PIabs, performance index. Data are expressed as mean ± SE (*n* = 3).

The wheat plants under low temperature had significantly lower net photosynthetic rate (Pn), stomatal conductance (Gs), maximum quantum efficiency of photosystem Ⅱ (Fv/Fm) and performance index (PIabs) than those under normal temperature (Figure 3, Table S1). The Pn and Gs of MT plants were significantly higher than that of N plants under low temperature. The chlorophyll a fluorescence parameters (i.e., Fv/Fm and PIabs) were significantly enhanced by MT under low temperature compared with the control.

**Figure 4:**
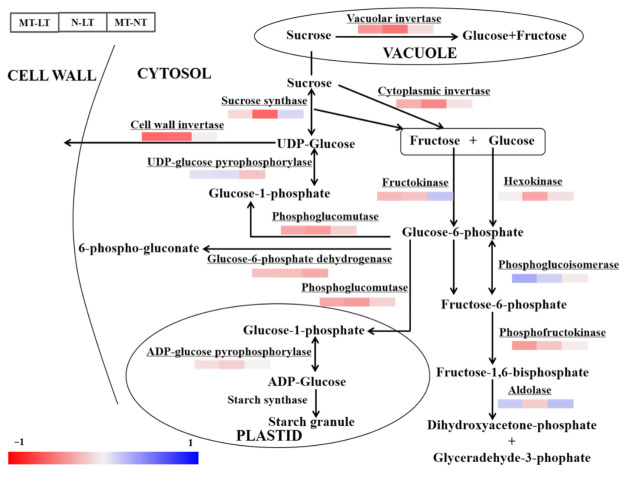
Heatmap of key carbohydrate metabolism enzyme activities in offspring wheat leaf as affected by parental melatonin treatment and low temperature. cwInv, cell wall invertase; vacInv, vacuolar invertase; cytInv, cytoplasmic invertase; UGPase, UDP-glucose pyrophosphorylase; PGM, phosphoglucomutase; PGI, phosphoglucoisomerase; G6PDH, glucose-6-phosphate dehydrogenase; FK, fructokinase; HXK, hexokinase; PFK, phosphofructokinase; Ald, Aldolase; AGPase, ADP-glucose pyrophosphorylase; Susy, sucrose synthase. The difference of activity for given enzyme among these treatments is deviation standardization and converted to a color scale. Increase and decrease in enzyme activity is indicated in the colorbar. MT, melatonin treatment; N, the control; LT, low temperature; NT, normal temperature. Data are expressed as mean ± SE (*n* = 3).

Compared with normal temperature, low temperature significantly reduced the activities of cytoplasmic invertase (cytInv), vacuolar invertase (vacInv), cell wall invertase (cwInv), phosphoglucomutase (PGM) and phosphofructokinase (PFK) in wheat seedlings (Figure 4, Table S1, Figure S3). However, low temperature significantly increased the activity of UDP-glucose pyrophosphorylase (UGPase). Under low temperature, the activity of Aldolase (Ald) was significantly enhanced by MT compared with the control. The activity of glucose-6-phosphate dehydrogenase (G6PDH) was reduced in MT plants in relation to the control.

## 3. Discussion

In the present study, the seed germination rate was markedly reduced by low temperature exposure, while both pre-soaking with melatonin and parental melatonin treatment significantly increased the germination rate in wheat under low temperature conditions. It has been shown that exogenous melatonin treatment alleviated damage of low temperature and improved the seed germination in cucumber [27]. Melatonin also enhanced the seed germination by osmotic regulation under salt stress in cotton [28]. It could activate the antioxidant systems to protect the seeds against drought-induced oxidative stress in wheat [29]. A similar result was found in *Phacelia tanacetifolia*, in which melatonin combined with priming medium significantly alleviated the negative effects of light and high temperature on seed germination [30]. Moreover, our previous study indicated that exogenous melatonin treatment on parental plants significantly increased the endogenous melatonin concentration in offspring germinating seeds [26]. During the seed germination phase, starch is hydrolyzed into soluble sugar with the involvement of amylase to provide energy. Low temperature decreases the activities of α-amylase and β-amylase, which hinders the degradation of seed stored starch [4]. However, seeds pre-soaked with melatonin had a higher starch degradation rate under low temperature stress, thus ensuring the energy supply and maintaining turgor pressure for the expansion of tissues during seed germination [31]. In addition, the germinating seeds from melatonin-primed maternal plants also showed accelerated starch degradation due to higher amylase activities under low temperature [26]. All this is consistent with the results in the present study.

Under low temperature conditions, the concentration of starch was significantly decreased while the concentration of soluble sugar was remarkably increased in germinating seeds both pre-soaked and parental treatment with melatonin compared with the control. It should be noted that melatonin stimulates root generation and vitality during germination and may have a positive effect on strengthening cucumber roots [32]. A similar result was found in rice under anoxic conditions [33]. However, there was no significant effect of melatonin treatments on the growth of radicle and coleoptile under low temperature, which might be due to the concentration effects of melatonin.

Both seed pre-soaking and parental treatment with melatonin significantly increased the activity of antioxidant enzymes while decreased the concentration of MDA in germinating seeds under low temperature. Low temperature-induced ROS accumulation could induce membrane lipid peroxidation [16]. MDA is a major product of membrane lipid peroxidation; on the contrary, its concentration is directly related to the extent of cell peroxidation [34]. Melatonin induces low temperature tolerance by reducing the MDA concentration in maize [31]. Antioxidant systems have key roles in prevent plants against oxidative damage [35]. For instance, the higher activities of SOD, CAT and APX in melatonin-primed barley plants help to mitigate the cell death induced by low temperature stress [18]. The foliar application of melatonin also results in the elevation of SOD activity in wheat under low temperature [21]. In agreement with this, our results indicated that the increased capacity of ROS scavenging could reduce the low temperature damage and be helpful for seed germination in melatonin-treated seeds under low temperature conditions.

Chloroplasts are easily affected adversely by low temperature, resulting in swollen and distorted thylakoids and less starch granules [36]. Melatonin could suppress the dark- induced senescence and chlorophyll degradation in barley leaves [37]. Under drought stress, the chloroplasts in melatonin-treated plants showed complete and clear edges, which cling to the cell wall [38]. Chloroplasts in melatonin-treated broccoli florets maintain a well-developed membrane system and the matrix layer of thylakoidsis was visible and arranged regularly [39], indicating that melatonin could promote the stress tolerance of chloroplasts [40]. In the present study, melatonin pretreatment alleviated the damage caused by low temperatures to the chloroplasts in the coleoptile, which should be beneficial to seed germination.

In the offspring, the Pn and Gs of seedling leaves were significantly increased by parental melatonin treatment under low temperature. Our previous studies have demonstrated that wheat plants treated with melatonin could maintain a relatively higher Pn and Gs compared with the control plants under low temperature conditions [19,26]. Melatonin has positive effects on photosynthetic rate, instantaneous and intrinsic water use efficiency, carbon fixation and starch synthesis [41]. Here, the Fv/Fm and PIabs of the offspring leaves were markedly improved by parental melatonin treatment under low temperature, indicating that melatonin alleviated the low temperature-induced inhibition of photoelectron transport in wheat. PIabs reflects comprehensively the number of active reaction centers and the electron transfer efficiency on the acceptor side, and it is sensitive marker to indicate the activity and state of PS II [42]. It has been documented that melatonin regulates the quantum yield of PS II donor and acceptor sides under low temperature [11]. The enhanced electron transport efficiency induced by parental melatonin treatment could be due to the reduced inhibition of electron flow at PS II oxidation sites [43], which benefited the low temperature tolerance of wheat seedlings.

In this study, parental melatonin treatment regulated the activities of carbohydrate metabolism enzymes under low temperature conditions. The activity of enzymes related to carbohydrate metabolism is the key factor in determining the physiological state and growth of crops [44]. Melatonin may play an important role in helping plants adapt to the later low temperature stress during cold acclimation [26]. It is well known that Ald catalyzes fructose-1,6-diphosphate to produce glyceraldehyde-3-phosphate that is involved in glycolysis and the pentose phosphate pathway (PPP) [45]. It has been shown that melatonin regulated the expression of glycolysis-related proteins in wheat under PEG stress, including Ald and hexokinase (HXK), and significantly increased the activity of nicotinamide adenine dinucleotide transporter, hence indirectly regulating the electron transport in the respiratory chain [41]. The G6PDH can represent the rate-limiting step of the pathway of carbohydrate alienation other than glycolysis is a key enzyme in PPP [46]. Its function is to provide reducing power (NADPH) and pentose phosphate for fatty acid and nucleic acid synthesis, which is involved in membrane synthesis and cell division [46]. Overexpression of *PsG6PDH* from *Populus euphratica* activated the expression of stress-related genes (including *NtERD10b*, *NtERD10c* and *NtSOD*) in tobacco plants, providing evidence for the regulatory function of *PsG6PDH* in plants under low temperature stress [47]. The activities of G6PDH is higher in melatonin-treated pomegranate fruit, maintaining sufficient intracellular NADPH supply [48]. Here, parental melatonin treatment increased the Ald and G6PDH activities of offspring seedlings under low temperature, indicating that melatonin modulated the sugar metabolism in offspring under such conditions. Thus, the parental melatonin treatment could improve the low temperature tolerance by regulating carbohydrate metabolism in offspring wheat, especially by modulating the activities of Ald and G6PDH.

## 4. Materials and Methods

### 4.1. Plant Materials and Treatments

#### 4.1.1. Seed Presoaking with Melatonin

Uniform seeds of winter wheat (cv. Jimai 44) were selected and disinfected with 1% sodium hypochlorite for 15 min, then washed with distilled water 7 times and dried with filter paper. The seeds were pre-soaked in 500 μM melatonin for 24 h. The concentration of melatonin was optimum based on the preliminary experiments with a range of 100, 200, 500, 1000 and 2000 μM melatonin concentrations (Figure S4). Seeds pre-soaked with distilled water were used as the control. Wheat seeds were provided by the Crop Research Institute, Shandong Academy of Agricultural Sciences (Jinan, China). Melatonin was purchased from Sigma-Aldrich (St. Louis, MO, USA).

Two pieces of filter paper were placed in each Petri dish with a diameter of 9 cm and equal volume of distilled water was added to soak the paper. Then, 50 pre-soaked seeds were put in each dish. Seeds pre-soaked with melatonin were cultivated at 12.0 ± 0.5 °C (MT). Half of seeds pre-soaked with distilled water was cultivated at 22.0 ± 0.5 °C as the control at normal temperature (CT1), and the other half was cultivated at 12.0 ± 0.5 °C as the control at low temperature (CT2). All seeds were germinated for 7 days in a dark incubator (Saifu Experimental Instrument Co., Ltd., Ningbo, China). After 7 days’ germination, seeds were sampled for analysis. A randomized block experiment design was used with three replicates per treatment.

#### 4.1.2. Parental Treatment with Melatonin

**Figure 5:**
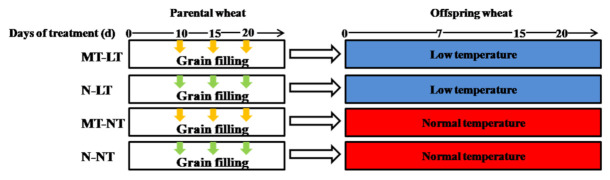
Schematic representation of experiment Ⅱ design. MT, melatonin treatment; N, the control; LT, low temperature; NT, normal temperature. Spraying with melatonin and water during grain filling stage is indicated with yellow and green arrow, respectively.

The parental melatonin treatment was conducted in a field trial at the Gongzhuling Experimental Station of Northeast Institute of Geography and Agroecology, Chinese Academy of Sciences (125.08° E, 43.72° N) in 2019. The same Jimai 44 winter wheat cv. was used. Two melatonin treatments were set: (1) 5 mM melatonin in the amount of 2000 L·ha^−1^ (MT) and (2) the control without melatonin (N). The amount of sprayed melatonin solution was determined according to the preliminary experiment. All sprays were carried out at dusk on the 10th, 15th and 20th day after anthesis. The randomized block experiment design was adopted with three replicates (plots) for each treatment. The plot size was 4 m × 2 m. Spike number per plant, grain number per spike, grain weight and grain yield per plant were determined at maturity. The harvested seeds were used for germination and seedling establishment experiments (Figure 5).

For the germination experiment, the harvested seeds that pretreated with melatonin and water parentally (MT and N) were disinfected with 1% sodium hypochlorite for 15 min, washed with distilled water seven times and dried with filter paper. The seeds were placed in a petri dish with two layers of filter paper, 50 seeds per dish. Half of the dishes of each type of seeds was cultivated at 12.0 ± 0.5 °C (LT), and another half was at 22.0 ± 0.5 °C as control at normal temperature (NT). The dishes were placed in the dark environment of incubator. Part of seeds were sampled for measurements after 7 days′ germination. The rest seedlings were moved to plastic containers (25 cm × 15 cm × 15 cm) in the Hoagland nutrient solution at 2-leaf stage [23]. Twelve plants were grown in each container. The growing temperatures were 12 °C/8 °C (day/night) for the low temperature treatment (LT) and 22 °C/18 °C (day/night) for control at normal temperature (NT), with a 12-h photoperiod at 22,000 Lux. A randomized block experiment design was used with three replicates per treatment. The latest fully expanded leaves were sampled for measurements at 4-leaf stage.

### 4.2. Measurements

#### 4.2.1. Germination Rate (GR), Length and Dry Weight of Radicle and Coleoptile

The germinated seeds of each dish were recorded to calculate germination rate (GR) at the 7th day of germination, which is the percentage of the number of germinated seeds to the total in each dish [49]. The lengths and dry weight of radicle and coleoptile were measured for ten seeds in each replicate.

#### 4.2.2. Extraction and Determination of Activities of Antioxidant Enzymes and Concentration of MDA.

The measurements of activities of antioxidant enzymes and MDA concentration were done according to Tan et al. [50]. Briefly, 0.5 g fresh samples were homogenized with 5 mL extraction buffer (50 mM phosphate buffer (pH 7.0) and 0.4% polyvinylpyrrolidone (PVP)) in a mortar on ice. The homogenate was centrifuged at 10,000× *g* for 30 min and the supernatant was used to determine activities of antioxidant enzymes and concentration of MDA.

The activity of SOD was determined by the nitroblue tetrazolium (NBT) light reduction method [51]. In brief, 3 mL reaction mixture consisted of 130 mM methionine, 750 μM NBT, 100 μM EDTA-Na_2_ and 0.05 mL enzyme extract in 50 mM phosphate buffer (pH 7.8). The reaction was started with 20 μM riboflavin by exposure to 4000 Lux light density for 10 min. The absorbance was measured at 560 nm and one unit of SOD activity was the amount of enzyme per g fresh sample required to inhibit 50% of the NBT reduction. The activity of CAT was determined by monitoring the decrease of absorbance at 240 nm [52]. Three mL reaction mixture consisted of 30% H_2_O_2_ and 0.1 mL enzyme extract in 200 mM phosphate buffer (pH 7.0). Changes in readings at 240 nm were recorded for 1 min after the starting H_2_O_2_ of the reaction at 10-s intervals. The activity of APX was determined by monitoring the decrease of absorbance at 290 nm [53].

Reaction mixture containing 20% trichloroacetic acid and 0.5% 2-thiobarbituric acid was added to 1 mL the suspension and heated to 100 °C for 20 min. Then the mixture was cooled and centrifuged at 4000× *g* for 10 min. MDA concentration was measured at 532 nm and corrected by subtracting the absorbance at 600 nm and 450 nm [54].

#### 4.2.3. Extraction and Determination of Sucrose, Soluble Sugar and Starch

One g of powdered dried germinating seeds was extracted thrice with 8 mL of 80% (*v*/*v*) ethanol at 80 °C for 30 min. Then, the mixture was centrifuged at 2000× *g*. The supernatant was collected to determine concentrations of sucrose and soluble sugar. The pellet was extracted twice with 9.2 M HClO_4_ at 100 °C using centrifugation at 2000× *g* to determine concentration of starch. The reaction mixture contained 0.9 mL supernatant extract, 2 M NaOH, 0.1% resorcinol and 10 M HCl was measured at 500 nm to determine the sucrose concentration. The reaction mixture contained 1.0 mL supernatant extract, 0.2% anthrone reagent and distilled water was measured at 620 nm to determine the soluble sugar concentration. Starch concentration was then measured following the same operation of soluble sugar concentration analysis described above [51].

#### 4.2.4. Extraction and Determination of Proline

Concentration of proline was measured following the ninhydrin method [55]. Briefly, 0.5 g fresh samples were mixed with 3% sulfosalicylic acid and heated at 100 °C for 15 min. After cooling, the mixture was centrifuged at 4000× *g* for 10 min. The supernatant was heated in boiling water bath for 15 min, and methylbenzene was then added after cooling. The methylbenzene layer was measured at 520 nm after keeping in the dark for layering.

#### 4.2.5. Transmission Electron Microscope (TEM)

Fresh sample (1 mm × 1 mm) was taken from the coleoptile center and operated on ice bed filled with 2.5% glutaraldehyde. Then, the sample was fixed with 2.5% glutaraldehyde, pumped using vacuum pump until the sample sunk to the bottom, and stored at 4 °C for determination. The treated sample was eluted, embedded in paraffin, sectioned and stained for transmission electron microscope (TEM) analysis (Hitachi HT7700, Tokyo, Japan) [56].

#### 4.2.6. Gas Exchange and Chlorophyll a Fluorescence

The Pn and Gs of the latest fully expanded leaves were measured LI-6400XT (LI-COR Co., Lincoln, NE, USA). The leaf chamber was equipped with 6400-02B red and blue light sources with 1200 μmol·m^−2^·s^−1^ during the measurement, and the CO_2_ concentration was 400 μmol·mol^−1^. Measurement was carried out from 10:00 _AM_ to 12:00 _PM_. Before measurement, leaves were acclimated in the chamber with 1200 μmol·m^−2^·s^−1^ light sources, ambient relative humidity, a temperature of 25 °C and a CO_2_ concentration of 400 μmol·mol^−1^. During the measurement, the leaf chamber kept stable. The Fv/Fm and PIabs were determined on the same leaf for gas exchange measurement by Fluorpen FP100 (Photon System Instruments, Drasov, Czech Republic) after a 30-min dark adaptation.

#### 4.2.7. Extraction and Determination of Carbohydrate Metabolism Enzymes

The key carbohydrate metabolism enzyme activities in leaf samples were determined following Jammer et al. [7], including UGPase, G6PDH, fructokinase (FK), HXK, PFK, Ald, ADP-glucose pyrophosphorylase (AGPase); sucrose synthase (Susy), PGM, phosphoglucoisomerase (PGI), cytInv, vacInv and cwInv. Briefly, 0.5 g fresh leaf samples were ground in liquid frozen and homogenized using 1 mL extraction buffer (40 mM Tris- HCl (pH 7.6), 3 mM MgCl_2_, 1 mM EDTA, 0.1 mM PMSF, 1 mM benzamidine, 14 mM β-mercaptoethanol, 24 μM NADP) and then incubated 30 min on ice. The homogenate was centrifuged at 13,200× *g* for 30 min under 4 °C. A fraction of the supernatant was dialyzed overnight with 20 mM potassium phosphate buffer (pH 7.4) at 4 °C for measuring the activities of FK, HXK, Susy, cytInv and vacInv. Non-dialyzed supernatant was used for determining the activities of UGPase, G6PDH, PFK, Ald, AGPase, PGM and PGI. The pellet was washed thrice with pre-cooled distilled water, resuspended in 1 mL high salt buffer (1 M NaCl, 40 mM Tris-HCl (pH 7.6), 3 mM MgCl_2_, 1 mM EDTA, 0.1 mM PMSF, 1 mM benzamidine, 14 mM β-mercaptoethanol, 24 μM NADP), and then mixed at 4 °C overnight. The homogenate was centrifuged at 13,200× *g* for 30 min under 4 °C, subsequently, the supernatant was dialyzed for overnight with 20 mM potassium phosphate buffer (pH 7.4) at 4 °C for measuring cwInv activity. The activities of cytInv, vacInv and cwInv were measured at 405 nm and the activities of UGPase, G6PDH, FK, HXK, PFK, Ald, AGPase, Susy, PGM, PGI were measured at 340 nm. The dynamics of absorbance at a given wavelength was monitored using an Epoch Microplate Spectrophotometer (Biotek, Bad Friedrichshall, Germany) with a 96-well microtiter format. The specific enzyme activity was analyzed within the linear range of substrate conversion, while was expressed in nkat g^−1^ FW.

### 4.3. Statistical Analysis

Data in experiment of seed presoaking with melatonin were subjected to the one-way analysis of variance (ANOVA) using the SPSS 22.0 (SPSS Inc., Chicago, IL, USA) to determine the differences between treatments. Data in experiment of parental treatment with melatonin were subjected to the two-way ANOVA to determine the effects of parental melatonin treatment, low temperature and their interaction. All data were firstly tested for homogeneity of variance before the ANOVA. For the heatmap representation, the difference of activity for given enzyme among the treatments is deviation standardization and converted to a color scale. Data are expressed as mean ± SE (*n* = 3).

## 5. Conclusions

Pre-soaking with melatonin increased the seed germination rate by enhancing antioxidant capacity and accelerating starch degradation under low temperature conditions, and alleviated the low temperature-induced damage to the chloroplasts in wheat seedling coleoptiles. Seeds from parental melatonin-treated plants had higher germination rate, elevated activity of antioxidant enzymes than the control seeds under low temperature conditions. In addition, parental melatonin treatment regulated the activities of carbohydrate metabolism enzymes, contributing to the enhanced low temperature tolerance observed in offspring wheat. It was suggested that both seed pre-soaking and parental treatment with melatonin could be the effective approaches for the induction of low temperature tolerance in wheat.

## Data Availability

The data presented in this study are available on request from the authors.

## Supplementary Materials

Table S1 Output of two-way ANOVA for the interactive effects of melatonin treatment and low temperature on the physiological traits in wheat. Figure S1 Effects of pre-soaked with exogenous melatonin on antioxidant enzyme activity and metabolite concentration after 7 days′ germination under low temperature in wheat. Figure S2 Effects of parental melatonin treatment during grain filling on antioxidant enzyme activity and metabolite concentration after 7 days′ germination of offspring wheat under low temperature. Figure S3 Effects of parental melatonin treatment during grain filling on key carbohydrate metabolism enzyme activities in the last fully expanded leaf of offspring wheat under low temperature. Figure S4 Preliminary study to determine the concentration of melatonin for pre-soaking wheat seeds.

## Sample Availability

Samples of the compounds are not available from the authors.
