# Supplementary material for: Induction of Low Temperature Tolerance in Wheat by Pre-Soaking and Parental Treatment with Melatonin
Source: Molecules. 2021 Feb 23;26(4):1192. doi: 10.3390/molecules26041192 (PMC7926940; doi:10.3390/molecules26041192)
Supplement: Supplementary file 1 [file molecules-26-01192-s001.pdf]

Table S1 Output of two-way ANOVA for the interactive effects of melatonin treatment and low temperature on the physiological traits in wheat

Germination	MT	LT	MT × LT	Seedling	MT	LT	MT × LT
Germination rate	**	**	**	Pn	*	**	ns
Radicle length	ns	**	ns	Gs	*	**	ns
Coleoptile length	ns	**	ns	Fv/Fm	**	**	*
Radicle dry weight	ns	**	ns	PIabs	ns	**	ns
Coleoptile dry weight	ns	**	*	cytInv	ns	**	ns
SOD	ns	*	ns	vacInv	ns	*	ns
CAT	ns	**	ns	cwInv	ns	*	ns
APX	ns	**	ns	UGPase	ns	*	ns
MDA	*	**	*	PGM	ns	*	ns
Sucrose	**	**	**	PGI	ns	ns	ns
Soluble sugar	ns	*	ns	G6PDH	*	ns	*
Starch	*	**	ns	FK	ns	ns	ns
Proline	ns	ns	ns	HXK	ns	ns	ns
				PFK	ns	*	ns
				Ald	*	ns	ns
				AGPase	ns	ns	ns
				Susy	ns	ns	ns

Note: MT, melatonin treatment; LT, low temperature. SOD, superoxide dismutase; CAT, catalase; APX, ascorbate peroxidase; MDA, malondialdehyde; Pn, net photosynthetic rate; Gs, stomatal conductance; Fv/Fm, maximum quantum efficiency of photosystem II; PIabs, performance index; cytInv, cytoplasmic invertase; vacInv, vacuolar invertase; cwInv, cell wall invertase; UGPase, UDP-glucose pyrophosphorylase; PGM, phosphoglucomutase; PGI, phosphoglucoisomerase; G6PDH, Glucose-6-phosphate dehydrogenase; FK, fructokinase; HXK, hexokinase; PFK, phosphofructokinase; Ald, aldolase; AGPase, ADP-glucose pyrophosphorylase; Susy, sucrose synthase. * and ** mean significant level of 0.05 and 0.01, respectively. ns means no significant difference.

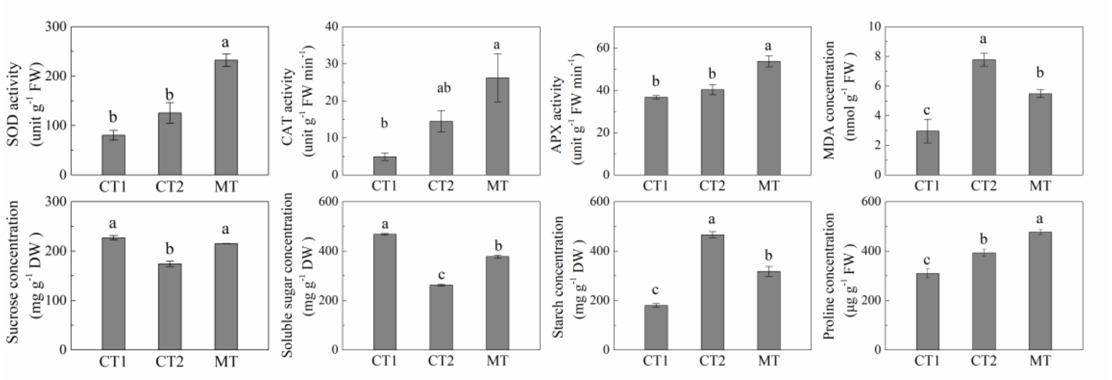

Figure S1 Effects of pre-soaked with exogenous melatonin on antioxidant enzyme activity and metabolite concentration after 7 days′ germination under low temperature in wheat. Different small letters mean significant difference between treatments at *P*<0.05 level. CT1, normal temperature control; CT2, low temperature control; MT, melatonin treatment under low temperature. SOD, superoxide dismutase; CAT, catalase; APX, ascorbate peroxidase; MDA, malondialdehyde.

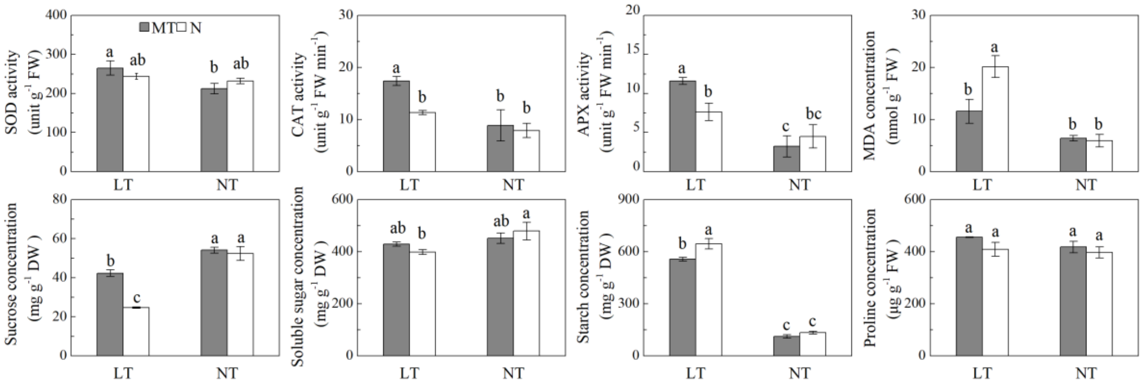

Figure S2 Effects of parental melatonin treatment during grain filling on antioxidant enzyme activity and metabolite concentration after 7 days′ germination of offspring wheat under low temperature. Different small letters mean significant difference between treatments at *P*<0.05 level. MT, melatonin treatment; N, the control; LT, low temperature; NT, normal temperature. SOD, superoxide dismutase; CAT, catalase;

APX, ascorbate peroxidase; MDA, malondialdehyde.

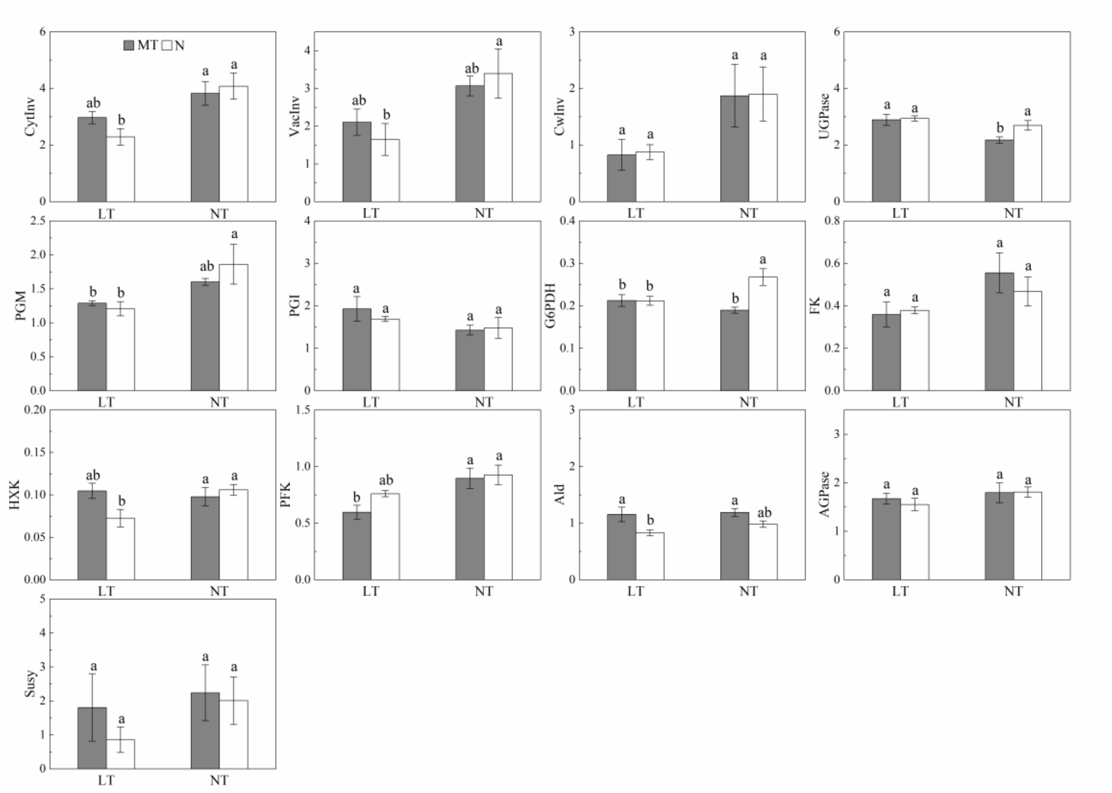

Figure S3 Effects of parental melatonin treatment during grain filling on key carbohydrate metabolism enzyme activities in the last fully expanded leaf of offspring wheat under low temperature. Different small letters refer to significant difference between treatments at $P< 0.05$ level. MT, melatonin treatment; N, the control; LT, low temperature; NT, normal temperature. CytInv, cytoplasmic invertase; VacInv, vacuolar invertase; CwInv, cell wall invertase; UGPase, UDP-glucose pyrophosphorylase; PGM, phosphoglucomutase; PGI, phosphoglucoisomerase; G6PDH, Glucose-6-phosphate dehydrogenase; FK, fructokinase; HXK, hexokinase; PFK, phosphofructokinase; Ald, aldolase; AGPase, ADP-glucose pyrophosphorylase; Susy, sucrose synthase.

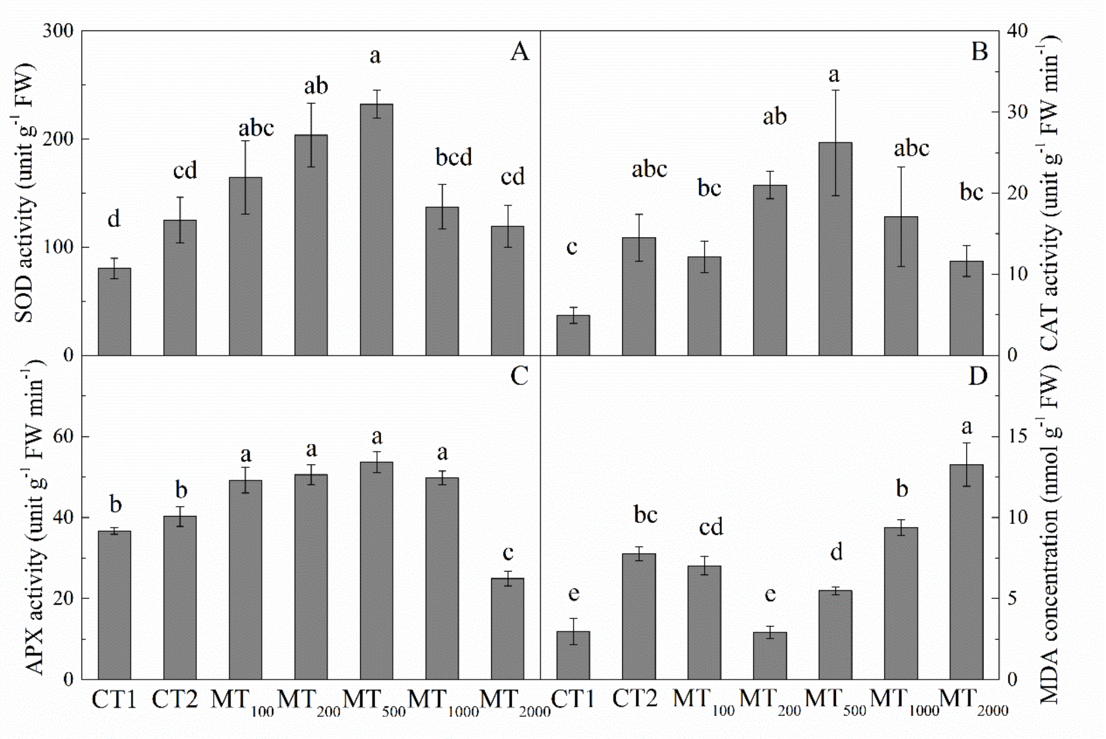

Figure S4 Preliminary study for determining the concentration of melatonin for pre-soaking wheat seeds. Effect of different concentrations of melatonin on the activities of antioxidant enzymes and concentration of MDA after 7 days′ germination under low temperature was presented. Different small letters mean significant difference between treatments at $P<0.05$ level. CT1, normal temperature (22 °C) control; CT2, low temperature (12 °C) control; MT_{100}, 100 μM melatonin treatment under low temperature; MT_{200}, 200 μM melatonin treatment under low temperature; MT_{500}, 500 μM melatonin treatment under low temperature; MT_{1000}, 1000 μM melatonin treatment under low temperature; MT_{2000}, 2000 μM melatonin treatment under low temperature. SOD, superoxide dismutase; CAT, catalase; APX, ascorbate peroxidase; MDA, malondialdehyde.
